# Spermatogenesis Beyond DNA: Integrated RNA Control of the Epitranscriptome and Three-Dimensional Genome Architecture

**DOI:** 10.3390/cimb48010123

**Published:** 2026-01-22

**Authors:** Aris Kaltsas, Maria-Anna Kyrgiafini, Zissis Mamuris, Michael Chrisofos, Nikolaos Sofikitis

**Affiliations:** 1 Third Department of Urology, Attikon University Hospital, School of Medicine, National and Kapodistrian University of Athens, 12462 Athens, Greece; ares-kaltsas@hotmail.com (A.K.); mchrysof@med.uoa.gr (M.C.); 2 Laboratory of Genetics, Comparative and Evolutionary Biology, Department of Biochemistry and Biotechnology, University of Thessaly, Viopolis, Mezourlo, 41500 Larissa, Greecezmamur@uth.gr (Z.M.); 3 Laboratory of Spermatology, Department of Urology, Faculty of Medicine, School of Health Sciences, University of Ioannina, 45110 Ioannina, Greece

**Keywords:** spermatogenesis, RNA-binding proteins, epitranscriptome, piRNA, noncoding RNAs, chromatin remodeling, 3D genome architecture, single-cell multi-omics, male infertility

## Abstract

Spermatogenesis is a tightly coordinated differentiation program that sustains male fertility while transmitting genetic and epigenetic information to the next generation. This review consolidates mechanistic evidence showing how RNA-centered regulation integrates with the epitranscriptome and three-dimensional (3D) genome architecture to orchestrate germ-cell fate transitions from spermatogonial stem cells through meiosis and spermiogenesis. Recent literature is critically surveyed and synthesized, with particular emphasis on human and primate data and on stage-resolved maps generated by single-cell and multi-omics technologies. Collectively, available studies support a layered regulatory model in which RNA-binding proteins and RNA modifications coordinate transcript processing, storage, translation, and decay; small and long noncoding RNAs shape post-transcriptional programs and transposon defense; and dynamic chromatin remodeling and 3D reconfiguration align transcriptional competence with recombination, sex-chromosome silencing, and genome packaging. Convergent nodes implicated in spermatogenic failure are highlighted, including defects in RNA metabolism, piRNA pathway integrity, epigenetic reprogramming, and nuclear architecture, and the potential of these frameworks to refine molecular phenotyping in male infertility is discussed. Finally, key gaps and priorities for causal testing in spatially informed, stage-specific experimental systems are outlined.

## 1. Introduction

Spermatogenesis is a highly orchestrated developmental process that underpins male fertility and ensures the faithful transmission of genetic and epigenetic information to the next generation. During spermatogenesis, diploid spermatogonial stem cells (SSCs) produce haploid spermatozoa through coordinated phases of mitotic amplification, meiotic recombination, and post-meiotic remodeling, ultimately leading to sperm maturation [1,2]. This program is controlled at multiple levels. Endocrine and niche-derived signals shape SSC maintenance and differentiation, whereas transcriptional and chromatin/3D-genome mechanisms govern meiotic entry, recombination, and MSCI; as transcription becomes restricted, post-transcriptional RNA regulation (RBPs, RNA modifications, and ncRNAs) assumes a progressively larger role through meiosis and spermiogenesis [3,4,5]. The fidelity of spermatogenesis is maintained through multilayered regulation that integrates systemic hormonal cues, local somatic cell support, and intrinsic germ cell programs [6].

Recent studies have particularly highlighted the central role of RNA-centered regulation, chromatin remodeling, and three-dimensional nuclear architecture [7,8,9]. The testis harbors a uniquely complex transcriptome shaped by significant cellular heterogeneity and the developmental decoupling of transcription from translation, supported by cytoplasmic messenger RNA (mRNA) storage and stage-restricted translational control [10,11,12]. Mature spermatozoa also carry molecular cargo beyond DNA, including retained nucleosomes at selected loci and noncoding RNAs (ncRNAs), such as microRNAs (miRNAs), long noncoding RNAs (lncRNAs), and PIWI-interacting RNAs (piRNAs). These components add further layers of post-transcriptional regulation and can modulate early embryonic development while transmitting paternal environmental information intergenerationally [13,14,15].

The growing recognition of these non-genetic contributions has profound implications for understanding male reproductive health, transgenerational epigenetic inheritance, and the pathogenesis of male infertility. Numerous studies have advanced our understanding of spermatogenesis; however, the literature remains fragmented across regulatory domains, with many studies focusing on isolated molecules, specific stages, or model systems. Recent advances in single-cell transcriptomics, RNA epigenetics, and nuclear architecture have reshaped the field, yet their mechanistic and integrative implications remain undersynthesized. Thus, this review adopts a mechanism-first perspective to examine the regulation of male germ cell development, highlighting insights from human and primate studies that clarify mechanisms or reveal species-specific features [15].

This review focuses on three major regulatory layers. Post-transcriptional control includes RNA-binding proteins and the epitranscriptome. Noncoding RNA-mediated regulation is examined with emphasis on the biphasic piRNA system and related noncoding RNAs. Chromatin dynamics and three-dimensional nuclear architecture coordinate transcriptional competence, epigenetic reprogramming, and genome packaging. Each section begins with a brief biological overview and concludes with key takeaways, aiming to provide a conceptual framework for understanding the principles and regulatory logic of spermatogenesis. Given the growing importance of understanding male germline regulation in both fertility and intergenerational epigenetic inheritance, this synthesis is both timely and essential.

## 2. Overview of Spermatogenic Cell Types and Molecular Transitions

**Figure 1:**
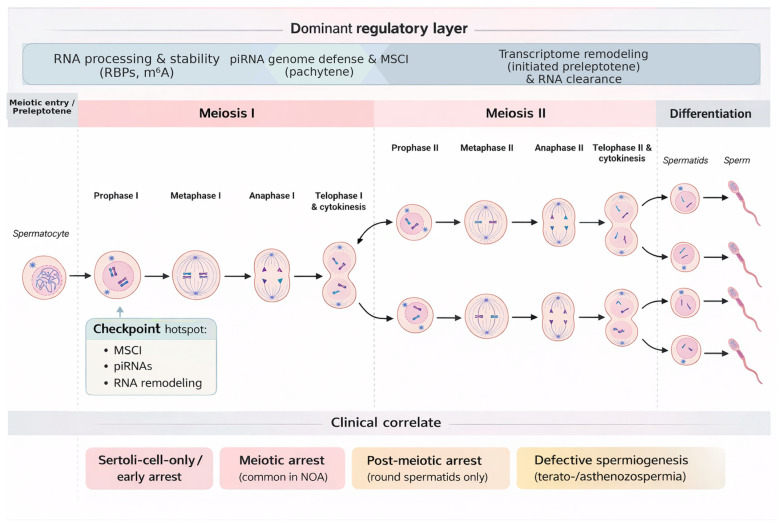
Stage-resolved spermatogenesis with dominant regulatory checkpoints and clinical correlates. Spermatogenesis is depicted from undifferentiated spermatogonia through meiosis I/II and spermiogenesis, highlighting a stage-specific “dominant regulatory layer,” defined here as the regulatory constraint that most strongly determines successful progression at that interval (e.g., SSC fate control, recombination–synapsis surveillance, MSCI-linked chromatin remodeling, or spermiogenic compaction with post-transcriptional timing). Transcriptome remodeling is initiated before meiosis (during differentiating spermatogonia and meiotic entry/preleptotene transition) and continues through pachytene and spermiogenesis, with increasing reliance on stored mRNAs, RBPs, and RNA modifications as transcription becomes progressively restricted. The meiotic checkpoint hotspot is placed in prophase I (pachytene), where synapsis and recombination completion converge with piRNA pathway activity and meiotic sex chromosome inactivation (MSCI). The lower band links failures to clinicopathologic outcomes (early arrest/Sertoli-cell-only pattern, meiotic arrest/non-obstructive azoospermia, post-meiotic maturation arrest, and defective spermiogenesis presenting as terato-/asthenozoospermia). Abbreviations: m^6^A, N^6^-methyladenosine; MSCI, meiotic sex chromosome inactivation; NOA, non-obstructive azoospermia; piRNA, PIWI-interacting RNA; RBP, RNA-binding protein; SSC, spermatogonial stem cell.

Spermatogenesis occurs through a continuous yet intricately organized sequence of developmental transitions, resulting in the formation of morphologically and molecularly distinct germ cell populations. This process is conventionally divided into three major phases: spermatogonial proliferation, meiotic division, and spermiogenesis, each characterized by specific cellular identities and distinct gene expression profiles [16,17]. A stage-resolved overview of dominant regulatory checkpoints and their clinicopathologic correlates is provided in Figure 1.

### 2.1. Spermatogonial Stem Cells and Differentiation

In the basal compartment of the seminiferous epithelium, SSCs sustain lifelong spermatogenesis by balancing self-renewal with commitment to differentiation. The defining checkpoint at this stage is the SSC fate decision, which must integrate local niche cues with intrinsic transcriptional programs to preserve an undifferentiated state while permitting periodic entry into differentiation. This transition also relies on RNA-centered regulation (selective mRNA stabilization, storage, and translation) and chromatin context, which together constrain when a spermatogonium becomes differentiation-competent. Clinically, disruption at this level most often presents as early spermatogenic arrest, severe oligozoospermia, or non-obstructive azoospermia (NOA) with basal-compartment pathology [18]. In rodents, undifferentiated spermatogonia are classically described as A_single, A_paired, and A_aligned based on clonal arrangement and intercellular bridges, with A_single cells representing a stem-enriched reservoir in steady state [19]. In humans, the classification is less clear, and the existence of a functionally distinct SSC population remains under investigation [19]. Traditionally, human and non-human primate spermatogonia have been histologically categorized into A_dark, A_pale, and B spermatogonia, based on nuclear morphology and staining patterns [20]. A_dark cells were historically considered reserve stem cells, while A_pale cells were viewed as actively proliferating SSCs.

However, this classification lacks definitive molecular markers, and recent transcriptomic studies suggest that human undifferentiated spermatogonia represent a heterogeneous population that cannot be easily mapped onto the classical morphology-based model [19,21]. SSC maintenance relies on niche-derived cues, including Sertoli-cell-derived growth factors such as glial cell line-derived neurotrophic factor (GDNF) and fibroblast growth factor 2 (FGF2) (with additional paracrine inputs shaping local self-renewal versus differentiation bias), and intrinsic regulators that safeguard the SSC program, including PLZF/ZBTB16 [18]. The transcriptional repressor PLZF (ZBTB16) is essential for self-renewal; PLZF-null mice progressively deplete the SSC pool and exhibit a complete loss of adult spermatogonia [22]. As aligned spermatogonia (A_aligned) commit to differentiation, they become retinoic-acid (RA)-responsive. Mechanistically, this SSC-to-differentiation transition provides a direct conceptual bridge to later sections because fate commitment is executed not only by transcriptional switches but also by stage-specific RNA processing and selective translation programs that preconfigure meiotic competence.

RA-dependent meiotic entry is the next major checkpoint, requiring precise timing of STRA8 and MEIOSIN induction and coordinated chromatin/RNA-state reprogramming to enable the mitosis-to-meiosis transition [23]. In the adult mouse testis, RA is produced in ~8.6-day pulses, which synchronize cohorts of differentiating spermatogonia along the tubules and contribute to the temporal coordination of spermatogenesis [24,25,26,27]. In contrast, similar periodic RA “waves” have not been observed in humans, suggesting that primate spermatogenesis may rely on tonic or non-cyclic cues to regulate SSC differentiation and meiotic initiation, highlighting significant species-specific differences [28].

### 2.2. Meiotic Spermatocytes

Following differentiation, preleptotene spermatogonia enter meiosis and become leptotene/zygotene spermatocytes in the adluminal compartment. The defining checkpoint here is successful execution of programmed DSB formation, repair progression, and homolog pairing/synapsis, which must be completed before cells can proceed through pachytene. These events are inseparable from higher-order chromosome architecture, because loop–axis organization and nuclear reconfiguration actively shape where breaks form and how recombination is resolved. Clinically, perturbation at this unit commonly manifests as meiotic arrest and is a major biological substrate of non-obstructive azoospermia [4,29].

Meiotic prophase I is a prolonged, checkpoint-dense interval in which chromosome structure is rebuilt to support recombination and quality control. During leptotene, chromosome axes assemble and chromatin loops are reorganized, establishing DNA double-strand breaks (DSB) competence and constraining break formation within loop–axis architecture; recombination intermediates then mature as cells progress through zygotene toward pachytene, in tight coupling with synaptonemal complex assembly [4,30].

The timing logic is therefore hierarchical: DSB formation must be followed by productive repair progression and completion of homolog synapsis, and failure at any step activates meiotic checkpoint networks that eliminate defective spermatocytes [29,31].

These prophase I architecture and checkpoint processes are foundational—not ancillary—because they directly condition later-stage phenomena discussed in this review, including meiotic sex chromosome inactivation (MSCI)/sex-body formation, piRNA-linked genome defense programs, and stage-dependent shifts toward post-transcriptional control as transcription becomes increasingly restricted in differentiating germ cells [32].

The histone methyltransferase PRDM9 directs DSB placement by depositing H3K4me3 at recombination hotspots; its loss results in defective homolog synapsis and mid-prophase arrest [33]. Then, as spermatocytes transition through zygotene and pachytene, homologous chromosomes pair and synapse, stabilized by the synaptonemal complex. Core components, such as SYCP1 and SYCP3, are essential for chromosomal alignment and structural integrity during these stages [34]. A subset of DSBs is repaired as crossovers, generating new allele combinations [35]. In mid-pachytene, the transcription factor A-MYB (*Mybl1*) is upregulated, driving a broad transcriptional program that extends into the post-meiotic phase [36].

MSCI is initiated during pachytene of meiosis I (not meiosis II), coinciding with completion of autosomal synapsis and persistence of largely unsynapsed X–Y regions that are sequestered into the sex body [32]. Due to the lack of full synapsis during pachytene, the sex chromosomes are recognized as unsynapsed chromatin and are compartmentalized into a distinct heterochromatic structure known as the sex body [32]. This process is initiated by the recruitment of the DNA damage response kinase ATR, which phosphorylates H2AX (γH2AX) across the XY domain. Concurrently, the sex body acquires repressive histone modifications such as H3K9me3 and macroH2A, reinforcing transcriptional silencing [37,38,39].

Disruption of MSCI, e.g., through defects in the ATR/TOPBP1 pathway, activates pachytene checkpoints, leading to spermatocyte apoptosis and meiotic arrest [40]. Conversely, failures in hotspot specification or synapsis cause pachytene arrest and hinder the formation of haploid cells [41]. After the completion of meiosis I, secondary spermatocytes rapidly enter meiosis II, a short division resembling mitosis that produces haploid round spermatids.

### 2.3. Haploid Round and Elongating Spermatids—Spermiogenesis

Post-meiosis, haploid round spermatids undergo spermiogenesis to form elongating spermatids and mature spermatozoa [2]. The defining checkpoint at this stage is successful execution of acrosome formation, flagellar assembly, nuclear shaping, and progressive chromatin compaction; failure yields post-meiotic maturation arrest or severe sperm structural defects. Mechanistically, spermiogenesis is distinguished by a strong reliance on post-transcriptional regulation because many transcripts required late are synthesized earlier, stored in RNP complexes, and selectively translated as transcription becomes progressively curtailed during chromatin condensation [5]. Recent work also supports phase-separated RNP assemblies as a mechanism for activating translation of stored mRNAs during late spermiogenesis, reinforcing the functional primacy of RNA-state control at this stage [42]. Clinically, disruption here most often presents as teratozoospermia and/or asthenozoospermia, and in severe cases as a round-spermatid–only phenotype.

Early haploid transcription is orchestrated by the germ-cell-specific transcription factor CREMτ, which activates key spermiogenic genes including protamines (Prm1, Prm2) and transition proteins (Tnp1, Tnp2) [43,44]. These proteins facilitate the progressive replacement of canonical histones, enabling the histone–transition protein–protamine exchange that drives extreme chromatin compaction. Protamines package DNA into a highly condensed toroidal architecture, which protects the paternal genome and establishes the hydrodynamic morphology of the sperm head [45].

Despite this global compaction, a minority of nucleosomes are retained at genes associated with early embryogenesis, development, and imprinting. These loci often harbor active or poised histone marks, suggesting their potential roles in shaping the epigenetic information delivered to the zygote [46,47]. As nuclear compaction intensifies, transcription becomes silenced, and elongating spermatids rely on the translational control of previously transcribed mRNAs. These transcripts are stored in ribonucleoprotein (RNP) granules and are translated in a stage-specific manner under the regulation of RNA-binding proteins, supporting processes including chromatin remodeling, acrosome biogenesis, flagellar assembly, and spermiation [48].

Together, these coordinated post-meiotic events produce mature spermatozoa that are structurally complete but functionally immature, requiring epididymal maturation to acquire motility and fertilization competence. A stage-resolved summary integrating RNA-centered regulation with chromatin and 3D genome remodeling across spermatogenesis is provided in Table 1.

## 3. RNA-Binding Proteins in Germ Cell Regulation

RNA-binding proteins (RBPs) are critical regulators of post-transcriptional gene expression and are broadly classified based on their subcellular localization. Nuclear RBPs act on nascent transcripts by coordinating pre-mRNA processing steps, including capping, polyadenylation, and splicing [49]. In contrast, cytoplasmic RBPs interact with mature mRNAs to regulate localization, stability, translation, and decay. Notably, some RBPs shuttle between cellular compartments and contribute to the export of mRNA from the nucleus [50].

During spermatogenesis, RBPs are abundantly and dynamically expressed, controlling gene expression timing as germ cells decouple transcription from translation [50]. This regulation is essential across all developmental stages, from spermatogonia to elongating spermatids, and includes also the processing of small RNAs such as piRNAs and miRNAs. The testis contains a large, stage-biased repertoire of RBPs (~1700 in mouse), and the loss of these factors typically leads to germ cell arrest at specific developmental transitions [7,51,52,53]. Human genetic studies also implicate RBP defects in idiopathic infertility [10]. Recent clinical-genetics and proteogenomic surveys further position RBPs as recurrent contributors to non-obstructive azoospermia and severe oligozoospermia, suggesting their potential as diagnostic markers and therapeutic targets [51]. Importantly, RBP function does not act in isolation.

Many RBPs operate within a layered regulatory system shaped not only by RNA sequence elements but also by chemical RNA modifications, known as the epitranscriptome [54]. Together, RBPs and the epitranscriptome form an integrated system that precisely orchestrates the temporal control of RNA fate in the male germline.

### 3.1. Representative RBPs and Mechanistic Examples

A diverse set of RBPs operates at critical stages of spermatogenesis to coordinate the stability, translation, and degradation of developmentally regulated transcripts. DAZL, a germ-cell-specific translational activator, facilitates the spermatogonial-to-meiotic transition by promoting the translation of pro-meiotic mRNAs, such as *Stra8*. *Dazl*-null males fail to initiate meiosis efficiently, reflecting its central role in the process [55]. In undifferentiated spermatogonia, IGF2BP1 (IMP1) stabilizes SSC-maintenance transcripts (*Lin28a*, *Dnd1*, *Gfra1*, *Mov10*), likely through cooperative interactions with ELAVL1 (HuR), which protect mRNAs from degradation. Notably, the loss of IGF2BP1 leads to early depletion of the SSC pool [56].

At meiotic entry, the N^6^-methyladenosine (m^6^A)-binding RNA helicase YTHDC2, together with MEIOC and the germ cell-specific RNA-binding protein RBM46, drives the mitosis-to-meiosis switch. These proteins bind to the 3′UTRs near U-rich motifs, promoting the degradation of mitotic mRNAs and enabling meiotic gene expression. The deficiency of *Ythdc2* or *Rbm46* leads to early meiotic arrest, highlighting the importance of transcriptome remodeling during this transition [57]. Importantly, recent research demonstrates that YTHDC2 is also essential during pachytene, where its absence leads to microtubule-dependent telomere clustering, significant transcriptome dysregulation, and apoptosis of spermatocytes. These phenotypes highlight its dual role in RNA turnover and the structural integrity of meiotic nuclei [58].

During prophase I, another RBP, the DEAD-box helicase DDX4 (MVH/Vasa) localizes to perinuclear germ granules, where it contributes to piRNA biogenesis and translational regulation. Mice lacking *Ddx4* exhibit transposon derepression, disrupted meiotic silencing, and arrest in early prophase [59]. Another critical RBP family in germ cells is the Tudor domain-containing (TDRD) proteins, which serve as scaffolds for the piRNA pathway. Members of this family, such as TDRD1, TDRD9, and TDRKH localize to germ granules and facilitate the biogenesis and function of MIWI- and MILI-bound piRNA complexes. These complexes are essential for transposon silencing and maintaining genomic stability during meiosis.

Mice lacking *Tdrd1* or *Tdrd9* exhibit derepression of transposable elements, meiotic arrest, and germ cell apoptosis, underscoring their indispensable role in the small RNA-based regulatory network of spermatogenesis [60,61,62]. In late meiosis and early spermiogenesis, YBX2 (MSY2) binds thousands of transcripts in round spermatids, forming mRNP storage granules that prevent premature translation. The loss of YBX2 leads to the precocious translation of protamines, impaired chromatin compaction, and male infertility [63]. Finally, the Pumilio family RBPs, PUM1 and PUM2, function as translational repressors by binding to conserved motifs in the 3′UTRs.

While single knockouts are largely compensated, a double knockout leads to spermatogenic failure, indicating that these proteins have redundant yet essential roles in maintaining post-transcriptional repression during germ cell development [64,65]. Thus, these representative RBPs illustrate the functional diversity of RNA regulatory mechanisms throughout spermatogenesis, and underscore how post-transcriptional control is finely tuned to developmental context and stage.

### 3.2. The Epitranscriptome: Writers, Erasers, and Readers Orchestrate Timing

Chemical modifications on RNA molecules add a dynamic regulatory layer to germ-cell gene expression. These reversible modifications fine-tune RNA metabolism by affecting mRNA splicing, export, stability, translation, and decay, often by recruiting or modulating RBPs. The most prevalent and well-characterized internal RNA modification, m^6^A, plays a critical role in spermatogenesis. It is deposited by the METTL3/METTL14 writer complex and removed by the demethylases ALKBH5 and FTO [66,67].

The testis exhibits a high abundance of these enzymes, and stage-specific m^6^A dynamics have been observed in transcripts that regulate the mitosis-to-meiosis switch and spermiogenesis. m^6^A exerts its effects primarily through YTH domain-containing “reader” proteins, which bind to methylated transcripts, modulating mRNA stability, localization, and translational efficiency [68,69]. Genetic studies demonstrate a stage-specific requirement for m^6^A regulation. Early germ-cell depletion of METTL3/METTL14 causes loss of m^6^A, aberrant persistence of mitotic programs, and failure to maintain undifferentiated spermatogonia. In contrast, later germ-cell deletion predominantly impairs spermiogenesis, leading to failed cytodifferentiation and mis-timed haploid gene expression.

Among m^6^A readers, YTHDC2 is uniquely positioned at the intersection of RNA modification and helicase-driven transcript remodeling. Together with MEIOC and RBM46, YTHDC2 facilitates the clearance of mitotic mRNAs and the establishment of meiotic gene expression at the leptotene/zygotene transition. Its inactivation triggers early meiotic arrest with defects in transcript turnover [69]. Additionally, ALKBH5, the testis-enriched m^6^A eraser, is essential for proper mRNA splicing and transcript stability during meiosis [70]. Its deficiency leads to abnormal spermatogenesis, a reduced sperm count, impaired sperm motility, smaller testicular size, and male infertility [71,72]. Additional epitranscriptomic marks complement m^6^A. m^5^C is deposited by NSUN family methyltransferases, particularly NSUN2 and NSUN7.

NSUN2 modifies tRNAs, mRNAs, and vault RNAs, influencing mRNA stability, translation, export and noncoding RNA processing [73,74]. Recent experimental evidence underscores the functional importance of m^5^C in spermatogenesis: NSUN2 localizes to the chromatoid body, a central cytoplasmic hub for RNA regulation in germ cells, and its absence prevents germ-cell differentiation in mice. In NSun2-knockout testes, germ cells arrest during early meiotic prophase (leptotene/zygotene), elongated spermatids and sperm are absent, and transcripts encoding small-RNA pathway components are significantly downregulated. This suggests that m^5^C methylation is not merely a passive mark but is actively required for the maintenance of post-transcriptional networks, small-RNA processing, and progression through meiosis [75].

Furthermore, pseudouridine (PUS enzymes) and A-to-I editing fine-tune coding potential and RNA structure during germ-cell remodeling. Although systematic stage-resolved maps in the human testis are still emerging, multi-omics integration suggests that RNA marks interface with RBP hubs to coordinate storage-versus-translation decisions in round and elongating spermatids, aligning protein supply with chromatin condensation, flagellum assembly, and spermiation [75]. Notably, the testis-enriched expression of *ALKBH5* and several m^6^A readers, combined with infertility phenotypes observed in knockout models, positions the epitranscriptome as a promising axis for both diagnostic and therapeutic exploration in idiopathic male infertility.

Future priority directions include the generation of stage-resolved, modification-specific epitranscriptome maps, systematic characterization of reader-specific targetomes, and functional rescue studies through modulation of writer or eraser activity in testicular organoid and explant systems [76,77].

### 3.3. RBP Interaction Motifs and System-Level Insights

RBPs rarely act in isolation. Instead, they recognize specific sequence or structural motifs, primarily in the 3′ UTRs of mRNA, and often function in combinatorial assemblies that regulate mRNA fate. These motifs serve as docking platforms that integrate regulatory inputs from multiple RBPs, small RNAs, and epitranscriptomic marks [78]. In germ cells, this motif-based control is particularly critical, as transcription and translation are temporally decoupled, necessitating precise coordination of mRNA storage, decay, and translation. Specifically, a testis-enriched “ER patch” (glutamate-arginine-rich motif) augments RNA binding by RBPs such as NONO. Precise deletion of NONO’s ER patch abolishes its RNA binding and disrupts the mitosis-to-meiosis switch, effectively phenocopying complete *Nono* loss [7].

Additional modular interactions act at distinct stages. CELF and MUSASHI family proteins regulate alternative splicing and polyadenylation. ELAVL and Hu proteins stabilize meiosis-essential mRNAs [56]. Tudor-domain RNA-binding proteins scaffold PIWI–piRNA complexes within germ granules to support piRNA biogenesis and transposon silencing [10]. Together, these networked, stage-biased activities form the post-transcriptional regulatory architecture of the male germline. In addition, chromatin-interacting RBPs engage nascent transcripts and local chromatin regulators to couple co-transcriptional splicing and transcriptional output with downstream mRNA fates, thereby extending RBP control across both nuclear and cytoplasmic compartments [53].

## 4. Noncoding RNAs in Spermatogenesis: Post-Transcriptional Architects of Germ Cell Development

Once considered “junk DNA”, ncRNAs are now recognized as key regulators of spermatogenesis, controlling gene expression at multiple levels [79]. Among these, miRNAs and piRNAs are particularly abundant and dynamically expressed in the testis. These small RNAs guide mRNA decay, translational repression, and transposon silencing through sequence-specific interactions and partnerships with RNA-binding proteins [9,80]. LncRNAs also play a role in chromatin remodeling, mRNA stability, and cytoplasmic granule function in germ cells [80]. Together, ncRNAs orchestrate stage-specific expression programs and safeguard genome integrity, supporting both spermatogenic progression and the epigenetic quality of gametes.

### 4.1. microRNAs (miRNAs) in Germline Regulation

In spermatogonia (including SSC-enriched states), meiotic spermatocytes, and post-meiotic spermatids, miRNA networks provide stage-biased post-transcriptional buffering that helps enforce orderly transitions despite rapidly changing transcriptional landscapes. MiRNAs are ~22-nucleotide noncoding RNAs that regulate post-transcriptional gene silencing by pairing with complementary sequences in target mRNAs, resulting in translational repression or degradation [81]. They are generated through a canonical biogenesis pathway that involves Drosha, Dicer, and Argonaute (AGO) proteins, all of which are highly expressed in the testis. The loss of these components severely disrupts spermatogenesis [82].

Testicular miRNAs exhibit dynamic, stage-specific expression patterns, with distinct subsets enriched in spermatogonia, spermatocytes, or spermatids. In contrast, several studies indicate that mature spermatozoa carry markedly fewer miRNAs, suggesting that miRNA-mediated regulation diminishes as germ cells differentiate into functional sperm [16,83]. Among the most well-characterized, the miR-34/miR-449 family is essential for spermiogenesis; their combined deletion impairs flagellum formation and chromatin condensation, with *CP110* identified as a target [82]. Other spermatid-enriched miRNAs, such as miR-18a and miR-469, also contribute to chromatin compaction and condensation during post-meiotic differentiation [84,85]. At earlier stages, miRNAs are crucial for spermatogonial maintenance and cell fate decisions [77].

For example, miR-21 supports SSC survival downstream of *ETV5*, while miR-202-5p inhibits premature differentiation by targeting *Dmrt6*; its knockout leads to accelerated meiotic entry, germline stem cell depletion, and misregulation of *STRA8* and *DMRT6* expression [86,87]. Similarly, miR-146 is enriched in undifferentiated mouse spermatogonia and inhibits RA-mediated differentiation of spermatogonia via repression of *Med1*, a coregulator of RA-receptors [88]. The let-7 family, expressed in spermatogonia and spermatocytes, promotes differentiation by targeting genes such as *Mycn*, *Col1a2*, and *Ccnd1* [89]. Beyond germ cells, many miRNAs are also expressed in Sertoli and Leydig cells, where they regulate their activity during spermatogenesis [90].

High-throughput profiling continues to uncover hundreds of dynamically regulated miRNAs across testicular cell types, including survival and cell-cycle regulators enriched in spermatogonia, as well as meiosis and remodeling regulators (e.g., miR-17-92, miR-34/449) in later stages [91]. It should also be noted that altered miRNA levels correlate with human infertility phenotypes (e.g., reduced miR-17-92 in Sertoli-cell-only syndrome; elevated miR-146 in maturation arrest), highlighting diagnostic potential, pending further functional validation [91].

### 4.2. PIWI-Interacting RNAs: Genome Guardians and Beyond in Male Germ Cells

In male germ cells, piRNA pathway activity occurs in developmental waves, with prominent functions in prospermatogonia and again during meiotic and post-meiotic stages to maintain genome integrity and shape stage-specific gene-expression programs. PiRNAs are a distinct class of small noncoding RNAs (~24–31 nt) that associate with PIWI-clade Argonaute proteins (MIWI/PIWIL1, MILI/PIWIL2, MIWI2/PIWIL4) and are expressed almost exclusively in the male germline [4]. Their best-established role is silencing transposable elements (TEs) in the fetal and postnatal testis, acting at both transcriptional and post-transcriptional levels, thereby preserving genomic integrity during epigenetic reprogramming [92]. PiRNAs are produced through two interconnected biogenesis pathways: primary processing and secondary (ping-pong) amplification [5,61]. In the primary processing, long single-stranded precursors from piRNA clusters are cleaved by PLD6 (Zucchini/MITOPLD), loaded onto PIWI proteins, 3′-trimmed by PNLDC1 (with factors such as EXD1), and 2′-O-methylated by HEN1 to ensure stability [5].

In prospermatogonia, the secondary “ping-pong” cycle amplifies TE-targeting piRNAs, as MILI and MIWI2 cleave complementary transcripts in an antisense-sense loop. This process is scaffolded by Tudor-domain proteins (e.g., TDRKH), while MOV10L1, a germline-specific RNA helicase, initiates processing [61,62]. In the postnatal testis, pachytene piRNAs dominate. These piRNAs are bound by MIWI (PIWIL1) in spermatocytes and round spermatids, arising from non-repetitive intergenic regions via the primary pathway, independent of ping-pong amplification. piRNA processing occurs on a mitochondrial platform composed of TDRKH, ASZ1/GASZ, PLD6, MOV10L1, and PNLDC1 [60].

Notably, MIWI’s N-terminal RG-rich motif and a PIWI-specific insertion module are essential for the efficient loading of pachytene piRNAs, including longer species implicated in mRNA translational activation and spermiogenic progression [63]. Reflecting its role in genome defense, the piRNA pathway evolves rapidly in response to transposon pressure. For example, rodents have acquired DNMT3C, a de novo DNA methyltransferase specifically dedicated to silencing TEs in the male germline, while new TE insertions can be assimilated into piRNA clusters across generations [64,65]. Regarding expression waves, piRNA expression follows a biphasic developmental pattern in the mouse testis, reflecting distinct biological functions at each stage.

The first wave, known as pre-pachytene piRNAs, emerges during fetal and perinatal stages and is enriched in transposon-derived sequences. Nuclear MIWI2 (PIWIL4) guides de novo DNA methylation via cofactors such as SPOCD1, while cytoplasmic MILI (PIWIL2) cleaves TE RNAs [93,94,95]. The loss of core components (e.g., *Mili/Piwil2*, *Miwi2/Piwil4*, *Mov10l1*) causes TE de-repression, DNA damage, meiotic arrest, and male infertility [96,97,98,99]. Human genetics mirror these findings, with biallelic variants in *PIWIL1*, *TDRD* genes, *DDX4*, *MOV10L1*, *PNLDC1*, and *SPOCD1* identified in cases of idiopathic azoospermia and meiotic arrest [11,100]. The second wave, comprising pachytene piRNAs, initiates around the zygotene–pachytene transition in meiotic spermatocytes and continues through spermiogenesis.

These piRNAs arise predominantly from large intergenic loci and, to a lesser extent, from 3′UTRs of protein-coding genes. Unlike pre-pachytene piRNAs, they generally lack TE complementarity and are not involved in ping-pong amplification [101,102]. Instead, these piRNAs regulate mRNA turnover, translational timing, and post-meiotic cytoplasmic remodeling. Functional studies demonstrate that disruption of pachytene piRNA biogenesis (e.g., in *MIWI* mutants) leads to spermiogenic arrest, despite intact LINE-1 silencing, indicating transposon-independent roles in germ cell differentiation [101,102,103,104]. Together, these two waves illustrate how piRNA function transitions from transposon defense in early development to gene regulatory roles in later spermatogenesis, highlighting the versatility of the piRNA pathway in germ cell maturation.

Beyond their well-established role in transposon suppression, piRNAs increasingly appear to regulate gene expression programs essential for spermatogenesis. In the postnatal testis, pachytene piRNAs orchestrate stage-specific mRNA regulation. piRNA–MIWI complexes can promote the translation of stored spermiogenic mRNAs in round and elongating spermatids through imperfect 3′UTR base-pairing, which recruits HuR (ELAVL1) and eIF3f; disruption of this pathway impairs acrosome biogenesis, and flagellum assembly, resulting in male sterility [103,105]. Additionally, nuclear PIWI–piRNA complexes contribute to timely mRNA clearance, as many meiosis and spermiogenesis transcripts accumulate abnormally in mutants [106].

Loss of piRNA pathway activity also activates p53-dependent stress checkpoints, leading to germ-cell apoptosis and increased sensitivity to DNA damage; Trp53 deletion can temporarily extend germ-cell survival but fails to restore fertility, underscoring the essential regulatory role of piRNAs beyond genome defense [105]. Recent work further illustrates the precision mechanisms governing piRNA function. A recent study described a “two-factor authentication” system for accurate methylation of evolutionarily young transposons: SPOCD1 engages SPIN1 at loci carrying dual H3K4me3/H3K9me3 signatures, licensing them for MIWI2-directed methylation and minimizing off-target epigenetic modification [105].

Building on this framework, C19ORF84 was identified as a molecular bridge linking PIWI–piRNA complexes to the DNA methylation machinery, reinforcing the transposon-silencing axis in male germ cells [100]. Finally, defective piRNA pathways have implications beyond infertility. Studies have shown that the loss of PIWI/piRNA pathway genes correlates with hypomethylation of transposable elements, such as LINE-1, which in turn may contribute to mutagenesis and chromosomal fragility in germ-cell-derived cancers [107]. Thus, the chronic inability to silence transposable elements promotes genomic instability, contributing to germ cell tumorigenesis and highlighting the importance of piRNAs in maintaining germline genome integrity [100].

### 4.3. Long Non-Coding RNAs

Across spermatogonia, meiotic spermatocytes, and round/elongating spermatids, lncRNAs act as stage-biased scaffolds for RNA regulation and chromatin-associated complexes, linking germ-cell identity to progression. LncRNAs, defined as transcripts longer than 200 nucleotides that lack protein-coding potential, have emerged as prominent regulatory molecules in the testis, where they act as scaffolds, guides, decoys, or cis-regulators [108]. The testis exhibits one of the most diverse and abundant lncRNA repertoires among tissues, with thousands of lncRNAs displaying stage- and cell-type-specific patterns throughout spermatogenesis, suggesting potential roles in meiotic progression, chromatin remodeling, and post-transcriptional regulation [80,109]. Although most lncRNAs in the testis remain functionally uncharacterized, there is growing evidence of their involvement in germline gene regulation.

Notably, studies indicate that a subset of lncRNAs, characterized by strong testis-specific expression patterns, appears to escape meiotic sex chromosome inactivation during the pachytene stage of meiosis, indicating that lncRNAs may play crucial roles in spermatogenesis [110]. Notably, Zhu et al. identified 1800 lncRNAs in human germ cells, with 157 exhibiting variable expression across several populations of testicular cells [111]. Among well-characterized examples, the lncRNA *Tcam1* (testis cell adhesion molecule 1-associated lncRNA) is expressed in pachytene spermatocytes and round spermatids, contributing to spermiogenic gene activation and particularly to immune response during spermatogenesis [112].

Another notable example is *Tesra*, an lncRNA expressed in male germ cells and shown to play significant roles during meiosis, especially in transcriptional activation. Disruption of *Tesra* affects key meiotic processes, indicating that lncRNAs can influence chromosomal events during germ cell development [113]. Additional factors with in vivo or cell-type evidence include *Rbakdn* (associated with meiotic progression), *Start* (involved in Leydig steroidogenesis and spermatocyte gene expression), *1700052I22Rik* (related to spermatid chromatin condensation), and *NLC1-C* (associated with spermatogonial proliferation and maturation-arrest status) [114,115].

LncRNAs also interact with chromatin regulators; although a definitive spermatogonial lncRNA that recruits PRC2 has yet to be identified, Sertoli-cell TUG1 binds to EZH2 and modulates H3K27me3 at *Ccl2*, illustrating Polycomb coupling in the testis [116,117]. Furthermore, *Tug1*-knockout male mice are sterile, exhibiting underlying defects, including a low number of sperm and abnormal sperm morphology [118]. Dysregulated lncRNAs have been increasingly linked to male infertility. Transcriptomic analyses of testicular biopsies reveal widespread lncRNA alterations across multiple infertility subtypes, with specific lncRNAs enriched in cases of immotile sperm or disrupted spermatogenesis [108].

Furthermore, a systematic review and meta-analysis identified several consistently deregulated lncRNAs implicated in key regulatory pathways such as apoptosis, cell cycle progression, and p53 signaling [119]. These findings not only highlight the functional relevance of lncRNAs in germ cell development but also nominate them as promising biomarkers for diagnosis and patient stratification in idiopathic male infertility. During spermatogenesis, non-coding RNAs form an integrated regulatory layer. PiRNAs safeguard the genome and choreograph germline gene expression. MiRNAs fine-tune stage transitions and structural remodeling. LncRNAs scaffold regulatory complexes and somatic–germline crosstalk. Disruption of these pathways perturbs timing, genome integrity, and differentiation, contributing to various forms of male infertility and testicular pathology.

## 5. Chromatin Remodeling and Epigenetic Modifications in Spermatogenesis

Male germ cells undergo one of the most dramatic and tightly regulated epigenetic reprogramming events in mammalian development. This includes the erasure and re-establishment of DNA methylation patterns during fetal stages, the dynamic deposition of stage-specific histone post-translational modifications (PTMs) throughout meiosis, and the histone-to-protamine exchange during spermiogenesis, which enables extreme nuclear compaction. These events are intricately coordinated with ATP-dependent chromatin remodeling complexes and 3D genome reorganization, ensuring the proper timing of gene expression, homologous recombination, and genome protection. Recent advances in single-cell epigenomics and multi-omics integration have begun to resolve the spatiotemporal specificity of these modifications in both mouse and human testes, revealing conserved principles as well as species-specific regulatory features [120]. Thus, this section reviews key epigenetic and chromatin-level events throughout spermatogenesis.

### 5.1. DNA Methylation Dynamics and Epigenetic Reprogramming

DNA methylation dynamics are most consequential during spermatogonial and early meiotic windows, when germ cells establish or maintain epigenetic states that condition later differentiation and influence sperm epigenome quality. DNA methylation is one of the most fundamental epigenetic mechanisms regulating gene expression during mammalian spermatogenesis. Male germline development is marked by two major waves of epigenetic reprogramming, which collectively reset, rebuild, and fine-tune the methylome to ensure germline identity and genomic stability [12]. During early development, primordial germ cells (PGCs) undergo near-complete demethylation as they migrate to the genital ridges [121]. This extensive erasure is essential not only for resetting epigenetic memory but also for removing aberrant methylation marks acquired during early embryogenesis. The second major wave of DNA methylation reprogramming occurs during spermatogenesis.

After the establishment of SSCs, de novo methylation begins in prospermatogonia and early spermatogonia through the coordinated action of the DNA methyltransferases DNMT3A, DNMT3B, and the regulatory factor DNMT3L [12]. Rodents have also evolved DNMT3C to target young retrotransposons, highlighting species-specific genome defense mechanisms [122]. By the onset of meiosis, most genomic regions in spermatocytes are remethylated, including transposable elements such as LINE-1, which require silencing to maintain genomic stability. Correct establishment of paternally imprinted genes (e.g., *H19*, *Igf2*, *Rasgrf1*) also occurs during this remethylation phase, leading to appropriate sex-specific methylation patterns [123].

However, recent genome-wide DNA methylation profiling across human spermatogenic stages has revealed that the methylome is not static after the early de novo methylation phase. Instead, it undergoes additional remodeling during meiosis and spermatogenic progression. Notably, primary spermatocytes exhibit a global but partial decline in DNA methylation, followed by progressive remethylation in post-meiotic spermatids. Hypomethylated regions are significantly enriched for transcription factor binding motifs of the DMRT and SOX families and overlap with spermatid-specific regulatory genes, indicating targeted hypomethylation at loci important for spermiogenic transcriptional programs [124]. Chromatin context also contributes to the establishment of the male-specific methylome.

Specifically, crosstalk between histone marks and DNA methylation is exemplified by NSD1-mediated deposition of H3K36me2, which recruits DNMT3A and counteracts Polycomb activity to ensure proper methylation patterning in prospermatogonia [125]. Disrupted spermatogenesis, often leading to male infertility, is marked by significant deviations from these programmed methylation trajectories [123]. Research has shown that disturbed spermatogenesis is associated with incorrect imprinting [126]. Furthermore, DNA methylation defects of *H19* and *MEST* within imprinted genes and *MTHFR* within non-imprinted genes have been associated with male infertility [12].

Studies also indicate that in disturbed spermatogenesis, germ cells exhibit considerable DNA methylation changes, which are significantly enriched in transposable elements and genes involved in spermatogenesis, highlighting the sensitivity of the germline methylome to pathological perturbation [124].

### 5.2. Stage-Linked Histone Modifications and Chromatin State Transitions

Stage-linked histone modifications are particularly prominent during meiotic prophase I (including the sex body during MSCI) and during the transition into spermiogenesis, where they coordinate transcriptional competence, repair/silencing programs, and preparation for nucleoprotein exchange. Chromatin structure in the male germline is dynamically remodeled through tightly regulated histone modifications that orchestrate germ cell differentiation, transcriptional transitions, and meiotic progression [127]. In spermatogonial cells, chromatin remains relatively open, with high levels of transcriptional activation-related H3K4 methylation marks (-me1, -me2, and -me3) that support spermatogonial stem cell maintenance and proliferation [128]. The onset of meiosis in spermatocytes triggers extensive chromatin reorganization. The histone methyltransferase PRDM9 catalyzes H3K4me3 and H3K36me3 at recombination hotspots, marking sites for programmed double-strand breaks and initiating homologous recombination [129,130]. During pachytene, autosomes retain transcription-linked marks, while the XY pair forms a sex body characterized by repressive PTMs during MSCI [131].

Throughout the leptotene, zygotene, and pachytene stages, dynamic patterns of histone methylation, including H3K9me1/2/3, H3K27me1/2/3, and H4K20me1/2/3, reflect the coordinated regulation of meiotic progression and genome stability [132]. As cells enter spermiogenesis, round spermatids undergo further chromatin remodeling, including localized enrichment of activating marks such as H3K4me3 and tightening of heterochromatin through repressive marks such as H3K9me3, which contribute to transcriptional regulation and transposable element silencing during post-meiotic development [133]. Early spermiogenesis also initiates molecular cues that mark the onset of chromatin remodeling toward histone eviction, most notably through histone hyperacetylation recognized by BRDT, setting the stage for the protamine-based compaction described in the next section [134,135].

Overall, the precise timing and distribution of histone modifications across spermatogonia, spermatocytes, and round spermatids are crucial for regulating meiotic progression, safeguarding genome integrity, and ensuring the successful execution of spermatogenic developmental programs. Studies show that abnormalities in histone-modifying enzymes or chromatin regulators at these stages can result in meiotic arrest, defective gene expression, and male infertility [127,136].

### 5.3. Histone-to-Protamine Transition and Nucleoprotein Exchange

The histone-to-protamine transition is executed in elongating spermatids during spermiogenesis and represents the key chromatin-compaction checkpoint required for sperm head shaping and genome protection. During spermiogenesis, male germ cells undergo one of the most extreme chromatin remodeling events in biology: the replacement of canonical histones with transition proteins and protamines. This process not only provides mechanical protection for the genome during transit but also facilitates the hydrodynamic shaping of the sperm nucleus for motility [45]. Before histones are replaced by protamines, the spermatid chromatin must become eviction-competent through a coordinated series of molecular events. This preparatory phase involves testis-specific histone variants and chromatin modifications that destabilize nucleosomes and recruit remodeling factors, creating a permissive landscape for nucleoprotein exchange [137].

To facilitate the histone-to-protamine transition, testis-enriched histone variants and chromatin modifications progressively destabilize nucleosomes and prime the genome for exchange. The linker histone H1T2/H1FNT accumulates in round and elongating spermatids, localizes to AT-rich heterochromatin, and contributes to chromocenter organization and nuclear shaping, which are essential for the asymmetric condensation of the sperm nucleus [138]. Core histone variants, particularly short H2A isoforms (e.g., H2A.B/H2A.L) weaken nucleosomal stability and promote histone displacement, thereby facilitating the transition to protamine-bound chromatin [139,140]. A critical eviction-competence axis links histone methylation with acetylation-dependent chromatin remodeling. NSD2-mediated deposition of H3K36me2 and H3K36me3 promotes recruitment of the acetyltransferase EP300, which catalyzes H4 acetylation at key residues.

These marks are then interpreted by BRDT, a testis-specific bromodomain protein that recognizes acetylated histone tails and is essential for histone eviction. Loss of *Nsd2* disrupts this cascade, resulting in reduced acetylation, histone retention, and defective chromatin compaction during spermiogenesis [141]. Despite near-global replacement, nucleosomes persist at selected promoters, architectural/centromeric sites, and other regulatory elements, often marked by H3K4me3/H3K27me3, suggesting a potential role in epigenetic inheritance and zygotic genome activation [142,143].

Clinical correlates: defective chromatin packaging in teratozoospermia. Because histone-to-protamine exchange is a principal driver of sperm nuclear shaping and compaction, protamine deficiency or impaired protamine incorporation can produce poorly condensed chromatin and head-morphology defects consistent with teratozoospermia [137]. In total and partial globozoospermia, higher rates of abnormal chromatin packaging and protamine deficiency have been reported compared with normozoospermic controls, supporting impaired nucleoprotein exchange as a mechanistically plausible contributor beyond the acrosomal defect [144,145]. These examples illustrate how late spermiogenic chromatin remodeling links molecular failure (packaging) to clinically recognizable morphology phenotypes.

### 5.4. ATP-Dependent Chromatin Remodeling Complexes

ATP-dependent remodelers act across meiosis and spermiogenesis, where they regulate chromatin accessibility, recombination-supportive architecture, and the execution of nucleoprotein exchange programs. ATP-dependent chromatin remodeling complexes play essential roles in shaping the germ cell epigenome by repositioning, ejecting, or restructuring nucleosomes, thereby regulating transcription, DNA repair, recombination, and chromatin compaction. These complexes use the energy of ATP hydrolysis to modulate nucleosome architecture and accessibility, and their functions are stage-specific and tightly regulated during spermatogenesis [146]. Among key remodelers, SWI/SNF assemblies, particularly those with BRG1 (SMARCA4), are essential for meiosis. Specifically, *Brg1* deficiency leads to infertility due to an arrest at meiotic prophase I in mice [147]. The PBAF subcomplex, which includes ARID2, functions during metaphase I, where it localizes to spindle checkpoint promoters and centromeres.

Loss of *Arid2* in the male germline results in spindle assembly defects and metaphase arrest, highlighting its critical role in chromosomal segregation [148]. The CHD family of remodelers plays temporally distinct roles: CHD4, as part of the NuRD complex, supports neonatal spermatogonia maintenance, while CHD5 becomes active in post-meiotic spermatids, facilitating terminal chromatin condensation. Notably, CHD5 dysregulation has been linked to human male infertility [149,150]. ISWI remodelers, such as SNF2H (SMARCA5), contribute to meiotic chromatin organization, including the formation of the sex body, and promote chromatin transitions leading to protamine incorporation during spermiogenesis [151].

Finally, chromatin remodeling is also necessary for recombination: HELLS (LSH) collaborates with PRDM9 at recombination hotspots to open chromatin and establish DSB competence, enabling proper homologous recombination during early meiosis [129].

### 5.5. 3D Genome Architecture Across Spermatogenesis

3D genome architecture shifts across the spermatogenic continuum, with major reorganization during meiotic prophase I and additional remodeling as spermatids undergo nuclear shaping and compaction. Genome-wide chromosome conformation capture studies (Hi-C, Micro-C) have revealed that the 3D chromatin architecture is extensively remodeled during spermatogenesis, reflecting the unique structural and functional demands of meiosis and sperm maturation. In mouse spermatocytes, as cells progress into prophase I, there is a progressive attenuation of topologically associating domains (TADs) and canonical loop/loop-domain structures; by pachytene, these conventional loop domains are largely lost, yet compartment-like A/B segregation remains discernible and continues to correlate with transcriptional activity [152,153,154]. Concomitant with loop/TAD loss, meiotic chromosomes adopt a loop-axis organization: linear chromosome axes from which large chromatin loops emanate.

Loop lengths increase over prophase I, ranging from ~0.8–1 Mb in early prophase to ~1.5–2 Mb in late prophase, consistent with increased chromatin compaction and the requirements for homolog pairing and recombination. Cohesin complexes (including meiotic-specific subunits) and axis components tether these loops, establishing a structural scaffold that aligns with homologous chromosomes under the synaptonemal complex and influences recombination site distribution and crossover formation [154,155]. Following meiosis, during spermiogenesis and in mature sperm, 3D genome architecture undergoes a second reorganization. A/B compartmental segregation and higher-order folding re-emerge in round spermatids and sperm, adapted to the extreme nuclear compaction characteristic of sperm chromatin [153,156].

However, classical loops and TADs are largely absent in sperm, revealing a fundamentally different chromatin topology compared to somatic cells [157]. Comparative analyses across species further suggest that germline-specific patterns of genome folding influence long-term genome evolution: regions subject to loop-axis remodeling often correspond to evolutionary breakpoints, indicating that recurrent 3D reprogramming in the germline may bias structural genome evolution over time [158]. For improved navigability across Section 4 and Section 5 and to minimize mechanistic dispersion, Table 2 summarizes each regulatory layer by predominant stage/cell type, representative effectors, primary function, and typical clinical correlate.

## 6. Insights from Single-Cell and Multi-Omics Studies

Single-cell and multi-omics technologies have transformed our understanding of spermatogenesis by elucidating cellular heterogeneity and developmental transitions with unprecedented precision. These approaches have mapped transcriptional and epigenetic programs across germ and somatic lineages, identified stage-specific regulators, and uncovered signaling interactions, aging effects, and species differences. This section highlights key insights from scRNA-seq atlases, integrated multi-omics studies, and comparative analyses that enhance our understanding of male germ cell regulation.

### 6.1. Single-Cell RNA-seq Atlases of Human and Mouse Testis

Recent single-cell transcriptomic atlases have precisely mapped the complete germ-cell continuum and associated somatic lineages in both human and mouse testes. These datasets encompass all major stages of spermatogenesis, from undifferentiated spermatogonia to elongated spermatids, as well as key somatic compartments, including Sertoli and Leydig cells [159]. Cross-species comparisons (human, macaque, mouse) from large-scale atlases have revealed that spermatogonia do not exist as a single pool but rather as multiple transcriptionally distinct states. A cross-species study identified six conserved spermatogonial states (SPG1–SPG6), reflecting a continuum from primitive stem-like cells to differentiating progenitors.

In humans, PIWIL4, MORC1, and TCF3 mark the most undifferentiated SPG subsets, while KIT marks cells committed to differentiation [13]. Pseudotime reconstruction across these atlases resolves the transition from mitosis to meiosis, charting the activation of STRA8/MEIOSIN programs, the onset of recombination, and the emergence of pachytene gene modules. These datasets also identify candidate MSCI escapees and highlight conserved versus primate-specific expression features, enhancing our understanding of species-dependent regulation of meiosis and spermiogenesis [13]. Additionally, a study of the human testis atlas reveals stable germ-cell trajectories throughout adult life, while somatic compartments exhibit greater transcriptional remodeling with age [159].

### 6.2. Integrated Multi-Omics and the Meiotic Demethylation Pulse

Recent single-cell multi-omics approaches, combining scRNA-seq, scATAC-seq, and single-cell DNA methylation profiling from the same cells, have generated high-resolution maps of transcriptional and epigenetic dynamics during human spermatogenesis. These studies demonstrate a strong coordination between stage-specific transcription and chromatin accessibility, particularly in SSCs, meiotic spermatocytes, and post-meiotic spermatids [120].

A striking finding is the discovery of a meiotic DNA demethylation pulse, characterized by a transient global loss of CpG methylation during early prophase I (leptotene–zygotene), especially at PRDM9-defined recombination hotspots. This pulse is conserved across species and is thought to facilitate chromatin remodeling and recombination. Experimental perturbation of this demethylation alters crossover frequency and distribution, supporting its functional role in meiotic progression [120]. Importantly, in some patients with NOA, leptotene spermatocytes retain DNA methylation at loci that are typically demethylated in healthy individuals. This suggests that the failure to undergo this epigenetic transition may represent a developmental checkpoint, and its disruption could contribute to meiotic arrest and infertility [120].

### 6.3. Cell–Cell Signaling and Age-Associated Changes

Ligand–receptor inference from human scRNA-seq revises niche architecture. CXCL12 is produced mainly by Leydig cells rather than Sertoli cells. CXCR4 is present on spermatogonia and macrophages, while CSF1R is macrophage-restricted, together suggesting a Leydig–macrophage–spermatogonia CXCL12–CXCR4 axis. Additional programs include NOTCH4/JAG1 in endothelium and persistent Hedgehog transcripts in myoid/Leydig cells [21]. Cross-species comparisons reveal conserved soma-to-germline signaling, including KIT and KITLG, alongside species-biased routes originating from Sertoli, peritubular myoid, and macrophage compartments [13]. Ageing primarily remodels soma and soma–germline interfaces: across >44,000 human cells, SSC numbers/transcriptomes are relatively stable, whereas Sertoli, myoid, Leydig, endothelial, and immune cells show metabolic, extracellular matrix (ECM), and inflammatory shifts; inferred SSC-directed signaling weakens and correlates with body mass index (BMI) in older men [160].

Recent datasets also indicate reduced base-excision repair in aged SSCs, oxidative-stress programs in Leydig cells (partially reversible ex vivo), and network-wide attenuation of pleiotrophin (PTN) signaling, consistent with remodeling rather than wholesale collapse of paracrine cues with age [161]. These soma-centric ageing shifts inevitably impact germ cells. Diminished retinoic acid signaling in the ageing niche, for example, may delay or desynchronize spermatogonial differentiation, as vitamin A metabolism in Sertoli cells becomes perturbed. Likewise, increased oxidative stress and pro-inflammatory changes in ageing Leydig and myoid cells can damage developing germ cells or trigger premature apoptosis [162].

Clinically, such cumulative niche dysfunction translates to poorer outcomes in older men—testes from advanced-age men more often show focal “Sertoli-cell–only” tubules and fewer spermatids, and men over ~40 with NOA have markedly lower sperm retrieval rates in testicular sperm extraction (TESE) procedures [162]. In essence, an ageing somatic environment yields a less supportive “soil” for the germline, contributing to the quantitative and qualitative decline in sperm production observed with advancing paternal age.

### 6.4. Cross-Species Comparisons and Inferred Regulators

Cross-species single-cell atlases from human, macaque, and mouse reveal a conserved transcriptional backbone for meiosis and spermiogenesis. However, they also identify notable species-specific features in early spermatogonia [13]. Comparative transcriptomic and epigenomic analyses further suggest that noncoding RNA programs diverge more rapidly across species than protein-coding genes, adding lineage-specific regulatory inputs to a conserved core [13,21,163]. Multi-omics integration has clarified the timing of regulatory events: chromatin opening precedes transcriptional activation at key promoters and enhancers during meiotic progression, while CREM-binding elements become accessible alongside the haploid transcriptional burst in spermatids [120]. Pseudotime analyses in mice nominate A-MYB (MYBL1) as a central switch during mid-pachytene, marking the onset of extensive transcriptional reprogramming [14,120].

These findings suggest that stage-specific transcription factor (TF) networks are established through coordinated changes in chromatin accessibility, rather than relying solely on mRNA expression. As multi-species datasets continue to grow, they offer critical opportunities to delineate conserved versus primate-specific regulators, and to investigate how species-divergent spermatogonial programs interface with a deeply conserved meiotic and post-meiotic machinery.

## 7. Literature Gaps and Future Directions

Despite substantial advances in understanding the epigenetic regulation of spermatogenesis, significant gaps remain regarding the timing, specificity, and functional consequences of epigenetic remodeling in germ cells. In the following section, we highlight the most critical unresolved questions in the field, as addressing these gaps is essential for elucidating the epigenetic determinants of male fertility and for advancing the development of reliable biomarkers and targeted therapeutic strategies.

### 7.1. Spermatogonial Stem Cell Regulation

While core niche-derived cues such as GDNF and FGF, along with intrinsic regulators like PLZF, have been implicated in SSC maintenance, the integration of these signals into a cohesive decision-making network governing human SSC self-renewal versus differentiation remains poorly defined. Most mechanistic insights arise from rodent models, yet primate SSC regulatory programs and soma–germline communication show notable species-specific differences, limiting the direct translation of findings to human biology [164]. Existing two-dimensional (2D) culture systems and xenotransplantation assays provide limited recapitulation of the native SSC niche.

However, emerging spatially informed co-cultures, 3D organoid and biomaterial platforms, and testis explant models, when combined with functional perturbation tools, offer promising opportunities to investigate candidate ligand–receptor interactions identified in single-cell atlases. In particular, these systems can be utilized to map how the spatial organization and molecular signaling of Sertoli cells, peritubular myoid cells, Leydig cells, and testicular immune cells shape SSC fate decisions [165,166]. Finally, comparative studies across species should prioritize human and non-human primate models to differentiate evolutionarily conserved mechanisms from lineage-specific adaptations. This approach has the potential to enhance translational strategies for male fertility preservation and restoration.

### 7.2. RNA-Binding Proteins

Comprehensive proteogenomic surveys have revealed a remarkably rich and stage-specific repertoire of RBPs in the testis, yet a substantial proportion remains functionally uncharacterized in vivo [10]. Several key gaps limit current understanding. For example, systematic functional validation (e.g., knockout, knockdown, and rescue experiments) is needed to pinpoint which RBPs are essential at discrete checkpoints, such as the mitosis-to-meiosis transition, MSCI, and spermiogenesis. Furthermore, the interplay among RBPs, whether cooperative or antagonistic, in regulating the localization, storage, stability, translation, and degradation of germline transcripts remains largely undefined.

There is also limited resolution of RBP targetomes across purified germ-cell states; techniques like CLIP-seq, RIP-seq, and integration with nascent transcription data (e.g., PRO-seq) will be instrumental for mechanistic dissection. From a translational perspective, only a limited number of RBP genes have been clearly linked to human male infertility, and many patient-derived variants remain of uncertain significance [10]. This highlights the need for scalable, functional screening platforms that connect clinical genetics with mechanistic insight. Finally, the impact of environmental exposures on germline RBP activity remains largely unexplored [15,167].

Combining toxicology models with RBP-centered transcriptomic and epitranscriptomic profiling could reveal post-transcriptional nodes that are sensitive to environmental stressors and relevant to fertility preservation.

### 7.3. Noncoding RNAs: miRNAs, piRNAs and lncRNAs

While research studies have cataloged thousands of germline-enriched ncRNAs, the field still faces significant gaps in mechanistic understanding, species-specific differences, and translational potential. At first, the pachytene piRNA program, which is dominant in meiotic and post-meiotic germ cells, remains mechanistically underdefined. Key unresolved questions include how individual piRNA species select their mRNA targets, whether their primary role is to promote mRNA decay, translation, or induce chromatin modifications, and how these activities are spatiotemporally coordinated throughout spermiogenesis [168]. The sensitivity of piRNA networks to heat, nutrient, or toxicant stress, along with their contribution to idiopathic maturation arrest, also warrant systematic investigation [168].

Furthermore, species-specific differences, such as the reliance on MIWI2 and DNMT3C for fetal DNA methylation in mice, both of which are absent in primates, limit extrapolation and reinforce the need for human- and primate-focused models to define conserved regulatory logic [168]. Testicular miRNAs and lncRNAs also show striking stage-specific expression, yet functional validation remains sparse. Although hundreds of candidates have been identified through profiling, only a few have been definitively linked to germ cell progression in vivo [169]. Future priorities include stage-specific perturbation of Dicer/Ago, systematic CRISPR interference/activation (CRISPRi/a) and knockout screens to test lncRNA function, and cross-species comparative studies to highlight primate-specific ncRNAs that may have relevance to humans.

Beyond their regulatory functions, ncRNAs are emerging as potential biomarkers for male infertility. Aberrant expression of specific sperm miRNAs, lncRNAs, and piRNAs has been associated with non-obstructive azoospermia, maturation arrest, and other subtypes of male infertility, suggesting potential utility in diagnostic panels or as indicators of germline stress and epigenetic risk transmission [9,91,119].

### 7.4. Chromatin Remodeling and Epigenetic Reprogramming

Core epigenetic transitions during spermatogenesis, including meiotic hotspot licensing, MSCI, histone-to-protamine exchange, and fetal de novo DNA methylation, are now understood in broad terms. However, the interconnected roles of histone modifications, DNA methylation, and nucleosome remodeling in enforcing stage-specific gene expression and developmental checkpoints remain incompletely defined. Emerging evidence places H3K36me2, deposited by NSD1/NSD2, as a key signal for targeting de novo methylation in the male germline, while remodelers such as HELLS and INO80 have been implicated in hotspot activation and XY chromatin silencing [142]. Despite these advances, the full writer–reader–remodeler circuitry that coordinates these processes, particularly its conservation in the human germline, remains to be elucidated.

Key questions persist regarding the genome-wide choreography of nucleosome eviction and selective histone retention during spermiogenesis, as well as the molecular regulators of protamine deposition and chromatin compaction. Addressing these gaps will require proteogenomic mapping in stage-purified germ cell populations, ideally paired with functional validation [142]. Finally, how paternal epigenetic information (retained histones, DNA methylation patterns, sperm RNAs) escapes post-fertilization reprogramming and influences offspring phenotype is a matter of ongoing debate [15]. Causal interrogation of epigenetic inheritance demands multi-omics profiling from sperm to zygote, controlled paternal exposures, and targeted epigenome editing in the male germline to separate correlation from mechanism [142].

### 7.5. 3D Genome and Multi-Scale Architecture

Hi-C and related chromatin conformation studies have revealed that TADs progressively attenuate during meiotic entry, giving way to axis-anchored loop arrays that support synapsis, recombination, and meiotic chromosome organization. These structures are subsequently replaced in haploid cells by a restructured 3D architecture, in which compartment features and some domainal organization are re-established despite extreme nuclear compaction in sperm [154,156]. However, how dynamic 3D transitions functionally interface with key processes, including recombination hotspot usage, MSCI fidelity, and transcriptional timing, remains incompletely understood. Critical questions persist regarding the mechanistic contributions of cohesin complexes, CTCF, and ATP-dependent remodelers in organizing meiotic and post-meiotic genome architecture at specific loci.

Emerging technologies now allow us to address these questions: CRISPR-based perturbations of architectural proteins, long-read multi-omics, and spatial genomics platforms provide opportunities to link structural features to functional outcomes across spermatogenic stages. Notably, comparative analyses suggest that germline-specific 3D reprogramming may influence evolutionary breakpoint landscapes, positioning male meiosis as a driver of genome evolution [170,171].

### 7.6. Single-Cell and Multi-Omics

Cross-species single-cell atlases have delineated the complete germ cell continuum and uncovered candidate regulators of fate transitions. However, dissociative approaches inherently disrupt spatial context, a limitation that can now be addressed by integrating spatial transcriptomics and chromatin profiling. Such approaches promise to reveal localized signaling niches and stage-defined cellular “neighborhoods” within the seminiferous epithelium that coordinate germline progression and soma–germline crosstalk [172]. Multi-omic profiling within the same cells has uncovered a conserved meiotic demethylation pulse, tightly linked to PRDM9 hotspot activity and promoter/enhancer activation at meiosis onset [172].

However, more comprehensive integration, encompassing RNA output, chromatin state, histone modifications, and proteome dynamics, is necessary to move from correlation to causality and to construct testable regulatory models of spermatogenic control. Closing this loop requires scalable and functional perturbation platforms. Tools such as CRISPRi/a, Perturb-seq in testis organoids or explants, and stage-specific conditional mouse models represent the next frontier for validating computationally inferred regulators and dissecting stage-specific gene regulatory networks.

## 8. Conclusions

Spermatogenesis is a highly orchestrated differentiation program in which spermatogonial stem cells generate mature spermatozoa through mitotic amplification, meiotic recombination, and post-meiotic remodeling, while maintaining genome integrity and developmental timing under a distinctive uncoupling of transcription from translation as chromatin progressively compacts. This review emphasizes that these transitions are governed by interconnected regulatory layers—post-transcriptional control by RNA-binding proteins and the epitranscriptome, noncoding RNA pathways, and chromatin/3D genome remodeling—that collectively coordinate cell-state progression and the establishment of a functional paternal genome.

Single-cell and multi-omics frameworks are now converting spermatogenesis from a stage-based description into a mechanism-resolved map, strengthening links between dysregulated RNA/chromatin programs and clinically relevant spermatogenic failure and supporting the integration of molecular phenotyping alongside histology. Key next steps include establishing human, stage-resolved RBP targetomes and RNA-modification landscapes, deploying causal perturbation strategies in organoid/explant and spatially informed systems, and integrating these datasets with human genetics and clinically annotated cohorts to prioritize actionable pathways and biomarkers. A systems-level view should refine the classification of spermatogenic arrest and help clarify how sperm chromatin and RNA cargo contribute to early embryogenesis and potential intergenerational effects.

## Figures and Tables

**Table 1:** Stage-specific integration of RNA-centered regulation with chromatin and 3D genome remodeling during spermatogenesis.

Spermatogenic Stage	Key RNA-Centered Regulatory Events	Coupled Chromatin/3D Genome Events	Consequences of Dysregulation
Undifferentiated spermatogonia/SSCs	mRNA stabilization and controlled translation RBP buffering of fate decisions (e.g., IGF2BP1, ELAVL1) RNA modification machinery supports stage-appropriate RNA fate (e.g., METTL3/METTL14)	Accessible chromatin at self-renewal programs Niche-dependent transcriptional competence	SSC depletion or premature differentiation Candidate molecular signatures in idiopathic infertility
Differentiating spermatogonia → preleptotene	Transcriptome shift toward meiotic competence Translational activation of pro-meiotic mRNAs (e.g., DAZL → STRA8 axis) Selective RNA turnover	Preparatory chromatin remodeling for meiotic entry Alignment of transcriptional output with impending recombination	Failure of meiotic initiation Differentiation defects contributing to NOA phenotypes
Leptotene/zygotene spermatocytes (meiotic entry)	m^6^A-linked transcriptome remodeling Decay of mitotic transcripts (e.g., YTHDC2–MEIOC–RBM46) Splicing/stability control (e.g., ALKBH5)	DSB formation at hotspots Early prophase nuclear reorganization begins	Early meiotic arrest Mechanistic basis for maturation arrest (histology–molecular integration)
Pachytene/diplotene spermatocytes	High-throughput RNA processing and surveillance piRNA pathway scaffolding and function (e.g., DDX4/MVH TDRD proteins); continued requirement for m^6^A-linked RNA remodeling (e.g., YTHDC2)	MSCI/sex body formation Attenuation of canonical loop/TAD organization with compartment-like features preserved (stage-resolved Hi-C)	TE de-repression, DNA damage, checkpoint activation → pachytene arrest Recurrent pathway in severe spermatogenic failure
Round spermatids (early spermiogenesis)	Physiologic uncoupling of transcription and translation mRNA storage in mRNPs Delayed translation timing (e.g., YBX2)	Initiation of histone remodeling Transcription progressively reduced as compaction programs begin	Mis-timed translation of spermiogenic effectors (e.g., transition/protamine programs) Defective differentiation → oligo-/azoospermia
Elongating spermatids → mature sperm	Final waves of translation and RNA clearance small-RNA cargo packaging Post-meiotic RNA regulation (e.g., PIWI–piRNA complexes)	Histone → transition protein → protamine exchange Extreme compaction with selective nucleosome retention at regulatory loci	Abnormal chromatin packaging, sperm structural defects, impaired fertilization competence Potential contribution to embryo-relevant epigenetic information

Abbreviations: SSC, spermatogonial stem cell; RBP, RNA-binding protein; mRNA, messenger RNA; mRNP, messenger ribonucleoprotein; m6A, N6-methyladenosine; DSB, DNA double-strand break; MSCI, meiotic sex chromosome inactivation; NOA, non-obstructive azoospermia; TAD, topologically associating domain; TE, transposable element; Hi-C, high-throughput chromosome conformation capture; PIWI, P-element–induced wimpy testis; piRNAs, PIWI-interacting RNAs; MVH, mouse vasa homolog; TDRD, Tudor domain–containing protein.

**Table 2:** Synthesis of major regulatory layers across spermatogenesis, organized by predominant stage/cell type, representative effectors, primary function, and typical clinical correlate.

Mechanism	Predominant Stage/Cell Type	Representative Effectors	Primary Function	Typical Clinical Correlate
SSC niche and fate control	Basal compartment: SSC/undifferentiated spermatogonia	GDNF, FGF2; PLZF/ZBTB16	Self-renewal vs. differentiation checkpoint	Early arrest; SSC depletion; NOA
RA-driven meiotic entry	Differentiating spermatogonia to preleptotene	RA; STRA8; MEIOSIN	Commitment to meiosis	Failed meiotic entry; differentiation arrest
Meiotic prophase I architecture and checkpoints	Leptotene to pachytene spermatocytes	Axis-loop organization; SPO11-DSBs; synaptonemal complex	DSB repair and synapsis surveillance	Meiotic arrest; NOA
MSCI/sex body	Pachytene spermatocytes	ATR pathway; γH2AX-associated silencing	Sex-chromosome silencing and quality control	Pachytene arrest
miRNAs	Spermatogonia to spermatids (stage-biased)	miRNA networks; AGO/RISC	Post-transcriptional buffering	Stage-specific dysregulation; abnormal sperm output
piRNAs	Prospermatogonia; pachytene spermatocytes; round spermatids	PIWI proteins; piRNA clusters	Transposon repression; germline RNA regulation	Arrest; spermiogenic defects
DNA methylation programming	Spermatogonia and early meiosis	DNMTs; TET enzymes	Epigenetic stability; imprint control	Infertility with epigenetic dysregulation
Histone PTMs	Prophase I and early spermiogenesis	H3K4/H3K9/H3K27 marks; writers/erasers	Chromatin-state transitions	Meiotic arrest; dysregulated programs
Histone-to-protamine exchange	Elongating spermatids	TNP1/2; PRM1/2	Nuclear shaping and compaction	Teratozoospermia; globozoospermia; DNA damage
ATP-dependent remodelers	Meiosis and spermiogenesis	SWI/SNF; CHD; INO80 complexes	Accessibility and packaging execution	Meiotic/spermiogenic failure
3D genome architecture	Across stages; major shifts in prophase I	Hi-C domains; loop extrusion; axis-loop organization	Nuclear organization and gene regulation	Misregulated progression; arrest phenotypes

Abbreviations: 3D, three-dimensional; AGO, Argonaute; ATR, ataxia telangiectasia and Rad3-related; CHD, chromodomain helicase DNA-binding protein; DNMTs, DNA methyltransferases; DSB, DNA double-strand break; GDNF, glial cell line–derived neurotrophic factor; FGF2, fibroblast growth factor 2; Hi-C, high-throughput chromosome conformation capture; MSCI, meiotic sex chromosome inactivation; miRNAs, microRNAs; NOA, non-obstructive azoospermia; PIWI, P-element–induced wimpy testis; piRNAs, PIWI-interacting RNAs; PLZF, promyelocytic leukemia zinc finger (ZBTB16); PRM, protamine; PTMs, post-translational modifications; RA, retinoic acid; RISC, RNA-induced silencing complex; SSC, spermatogonial stem cell; SWI/SNF, SWItch/Sucrose Non-Fermentable; TET, ten–eleven translocation; TNP, transition protein; γH2AX, phosphorylated histone H2AX (Ser139); INO80, INO80 chromatin remodeling complex.

## Data Availability

No new data were created or analyzed in this study. Data sharing is not applicable to this article.
